# Balance, gait, functionality and strength: comparison between elderly fallers and non-fallers

**DOI:** 10.1590/bjpt-rbf.2014.0085

**Published:** 2015-04-27

**Authors:** Elaine C. Cebolla, André L. F. Rodacki, Paulo C. B. Bento

**Affiliations:** Departamento de Educação Física, Centro de Estudos do Comportamento Motor (CECOM), Universidade Federal do Paraná (UFPR), Curitiba, PR, Brazil

**Keywords:** falls, aging, gait, functionality, muscular strength, movement

## Abstract

**BACKGROUND:**

Accidental falls are a major health problem related to aging and affect one in every three elderly individuals over the age of sixty.

**OBJECTIVE:**

To evaluate and compare the muscle strength, gait kinematics parameters, and performance in functional tests between elderly subjects with and without a prior history of falls. In addition, the association between the history of falls and the variables that demonstrated differences between groups were tested.

**METHOD:**

62 elderly subjects participated in the study and were allocated to the group with falls history (FG; n=20; 68.0±6.9 years old) or the group without falls history (CG; n=42; 65.5±4.1 years old). Maximal strength, gait kinematics parameters, and functional tests were tested.

**RESULTS:**

The FG showed lower muscle strength in the knee flexors (51.45±8.6 vs. 62.09±19 Kg), lower average toe clearance during the swing phase (0.04±0.006 vs. 0.043 ± 0.005 m), and lower performance in the "8-foot up-and-go" test (5.3±0.7 vs. 5.8±0.7 s) (p<0.05). There were no associations between any variables and falls, but the increased time in the "8-foot up-and-go" test may double the likelihood of a fall occurring.

**CONCLUSION:**

Fallers have reduced lower limb strength, gait alterations, the worst performance in the dynamic balance test, and an increased risk of falls.

## Introduction

Accidental falls are a major health problem and affect one in every three elderly individuals over the age of 60. The rate of occurrence of falls increases with age and may affect 45% of the elderly population over the age of 75^1^. The consequences are functionality reduction, loss of independence and, in some cases, may result in death. Moreover, falls lead to increased healthcare costs and social problems^2^^,^ 3.

The occurrence of falls depends on extrinsic factors (related to the environment) and intrinsic factors (related to the subject). Strength and power reduction, postural control and gait parameter alterations, as well as visual, functional, and cognitive deficits^1^^-^ 5 have been listed as the main intrinsic factors.

Elderly subjects sustaining one fall^6^ during the last year showed a greater center of pressure displacement in the medial lateral direction than non-fallers^7^. The drop in muscle strength due to aging is closely related to falls during locomotion^8^^,^ 9. In addition, a lower rate of torque development in the knee flexor muscles has been reported among fallers^10^, which indicates that muscle force-generating properties are relevant. Indeed, the peak hip, knee, and ankle torques reported for a single-step balance recovery from a lean angle of 10 degrees were approximately 150, 75 and 50 Nm, respectively, while peak torques required for the successful recovery after a trip range from 50 to 200 Nm^11^. These peak forces and torques may be difficult for some older adults to achieve^12^. A worse performance in the functional tests has been also reported^13^. However, studies that evaluated muscle strength, gait, and functionality did not assess the contribution of all these variables to fall occurrence in the same investigation. Differences in these parameters between samples may lead to discrepant results when considered together as they may influence each other.

The purpose of this study was to determine and compare the dynamic strength of lower limb, kinematic gait parameters, and functional test performance among elderly subjects with and without fall history. Furthermore, the association between fall history and the variables that showed significant differences between fallers and non-fallers was tested.

## Method

### Sample

A power analysis was calculated retrospectively and showed that, with 20 subjects in the fall group and 42 subjects in the control group for the 8-foot up-and-go test and considering an alpha level of 0.05, the power was 65% and the effect size was 0.5. Although most studies have been conducted with a similar sample size, it may be viewed as a limitation of the present study. We chose the 8-foot up-and-go test to calculate the sample size based on previous studies that revealed the sensibility of this test to discriminate elderly fallers^14^. The power analysis was calculated using the software Statistica (Statsoft, version 7.0).

Sixty-two elderly subjects (13 men and 49 women) recruited from local community volunteered to participate in the study. The inclusion criteria were: aged 60 years old or above, able to perform the activities of daily living and to walk independently, free from recent orthopedic problems such as surgery, fractures or other health problems that would prevent them from performing the physical tests. All procedures were approved by the Ethics Committee of Universidade Federal do Paraná (UFPR), Curitiba, PR, Brazil, under number 0835.0.000.091-10, and all subjects signed a consent form before participating, according to National Health Council Resolution 196/96.

The subjects attended six laboratory sessions to perform all evaluations. Initially, all subjects underwent a clinical examination and answered a customized questionnaire before performing the physical tests. The questionnaire was designed to identify whether the subjects had experienced a fall during the last 12 months. A fall was defined as unintentionally coming to rest on the ground, floor or other lower level, whether or not it produced an injury^15^. According to the questionnaire outcomes, subjects were allocated into one of two groups: fallers (FG; n=20) and non-fallers (CG; n=42).

### Dynamic strength measurements

After two familiarization sessions, one maximal repetition test (1RM) was performed to assess the dynamic strength of the lower limbs. The exercises were performed on three different exercise machines: leg-press, knee extensor, and flexor (Nakagym^(r)^, Brazil). Tests were executed in a random order and included concomitant movements of both lower segments. These exercises are chosen based on the relevance of the respective muscle groups during the performance of daily life activities such as climbing stairs and standing up from a chair^16^.

After a 10 min warm-up period on the treadmill at 5 Km/h, participants performed five trials to determine the maximal load. Verbal encouragement was provided during the tests and a 2 to 5 min resting period was imposed between trials.

### Gait evaluation

**Figure 1.**
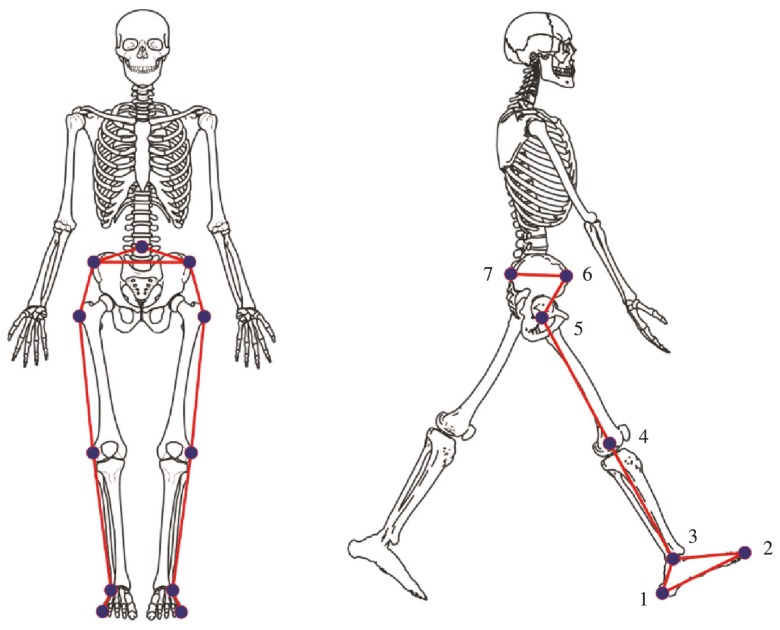
Biomechanical model to gait analysis. 1 - Calcaneus; 2 - Base of the 5th metatarsal; 3 - Malleolus side; 4 - Epicondyle of the femur; 5 - Greater trochanter; 6 - Anterior superior iliac spine; 7- 5th Lumbar vertebra.

The kinematics analysis was conducted to determine the spatial and temporal gait parameters related to falls^17^. Gait assessment was performed on a level surface in the laboratory's data collection area. A motion capturing system (Vicon Peak, Oxford, UK), consisting of six MX13 infrared cameras sampling at 100 Hz, identified the position with a set of markers placed over the skin and clothes (Lycra shorts). The coordinates of these markers were filtered (quantic spline) and used to reconstruct the movement in 3D by applying the biomechanical model (Figure 1). The subjects were instructed to perform ten trials, walking at volitional speed along an 8 m long and 2 m wide walkway. An intermediate gait cycle record was selected for analysis purposes. The calibration was performed according to manufacturer recommendations and yielded a volume of 3.0 m long, 1.5 m wide, and 1.7 m height. Three valid cycles were selected for digitizing purposes and a gait cycle was defined as two consecutive heel strikes. The gait cycle was time-normalized (0 - 100%) and the ensemble average of three valid trials was calculated. The variables analyzed were: mean speed (m.s^-^ 1), i.e. the product of stride length and cadence; stride time (s) - time from initial to final foot strike of the right foot; stride length (m) - the distance between initial and final foot strike of the right foot; cadence (stride·s^-^ 1) - number of strides per minute; speed of heel contact (m.s^-^ 1) - the vertical velocity of the heel marker in the vertical direction during the contact instant; and toe clearance - the smallest distance between the base of the 5^th^ metatarsal phalange and the ground during the mid-swing phase.

### Functional testing

The participants attended one session to perform a series of functional tests that included the 6-min walk (6MW) test, the sit-and-reach test, the 8-foot-up-and-go test, and the 30-s chair-stand test^18^.

The test sequence was performed in the following order: 1^st^ day - fall questionnaire; 2^nd^ and 3^rd^ days - strength test familiarization (1RM) and functional tests; 4^th^ and 5^th^ days - strength test (1 RM - 2 exercises in the 4^th^ day and 1 on the 5^th^ day); 6^th^ day - gait evaluation.

### Statistics

Data normality and group homogeneity were confirmed using the Kolmogorov-Smirnov and Levene tests, respectively, and the groups were compared using an independent "t" test. The data that did not present normal distribution were tested using non-parametric tests (the Mann-Whitney U test). A multivariate logistic linear regression was used to verify the association between falls and all variables that presented statistical differences between fallers and non-fallers. Variables highly correlated were determined and only one was included as input in the regression analysis. All statistical analyses were performed using Statistical Software (StatSoft, version 7, USA), and the level of significance was set at p<0.05.

## Results

No differences were found in age and physical characteristics between the CG and FG (Table 1).

**Table 1.** Physical characteristics of the participants (mean ± standard deviation).

Variable	CG (42)	FG (20)	P
Age (y)	65.5±4.10	68.0±6.90	0.18
Body mass (Kg)	75.1±14.30	77.1±11.70	0.58
Height (cm)	160.1±9.40	155.9±4.60	0.20

CG: control (non-fallers); FG: fallers; (p>0.05).

The FG showed lower dynamic strength in the knee flexor muscle than the CG (p<0.01). No differences were found between groups for knee extensor (p=0.06) and leg-press exercise (p=0.30) (Table 2).

**Table 2.** Dynamic strength of the lower limb (mean ± standard deviation).

	Exercises	CG	FG	P	ES
	Knee extensor	65.64±19.77	58.15±11.28	0.06	0.42
1 RM	Knee flexor	62.09±19.33	51.45±8.65	<0.01*	0.63
	Leg-press	67.15±17.31	64.3±8.71	0.30	0.20

ES: effect size; 1 RM: one-repetition maximal;

* (p<0.05).

The spatial and temporal gait parameters are shown in Table 3. The FG showed lower minimum toe clearance during the gait swing phase than the CG (p=0.04), and no further differences were found in other kinematic variables.

**Table 3.** Spatial and temporal gait parameters (mean ± standard deviation).

Variables	CG	FG	P	ES
Stride length (m)	1.17±0.17	1.11±0.12	0.1	0.38
Stride time (s)	1.08±0.09	1.07±0.08	0.7	0.11
Cadence (strides/s)	0.93±0.08	0.93±0.06	0.8	0
Gait speed (m/s)	1.08±0.18	1.04±0.16	0.3	0.23
Toe clearance(m)	0.043±0.001	0.040±0.001	0.04*	0.003
Heel contact (m/s)	0.76±0.36	0.70±0.31	0.4	0.17

ES: effect size;

* (p>0.05).

The FG had a worse performance in the 8-foot up-and-go test when compared to the CG (p=0.02). There were no differences between groups for the 30-s chair-stand, sit-and-reach, and 6MW tests (p>0.05) (Table 4).

**Table 4.** Functional tests (mean ± standard deviation) of the faller (FG) and non-faller group (CG).

Variables	CG	FG	P	ES
30-s chair-stand (rep)	13.14±2.48	12.35±2.34	0.2	–0.32
Sit-and-reach (cm)	–3.39±12.22	–2.28±9.33	0.7	0.09
8-foot up-and-go (s)	5.37±0.75	5.84±0.73	0.02*	0.63
Six minute walk (m)	571.53±67.51	542.93±66.69	0.1	–0.42

ES: effect size;

* (p>0.05).

The logistic regression analysis was performed to assess whether knee strength flexors, minimum toe clearance or performance in the 8-foot up-and-go test were associated with falls. The results showed no association between knee flexor strength and falls (β, -0.02, p=0.20, odds ratio 0.97, CI 0.93-1.02) or between minimum toe clearance and falls (β, -88.5, p=0.09, odds ratio 0.00, CI 0.00-infinity). Falls and the time to perform the 8-foot up-and-go test presented a non-significant, but borderline trend between groups (β, 0.83, p=0.06, odds ratio 2.30, CI 0.95-5.59).

## Discussion

The similar anthropometric characteristics between groups allowed comparisons without confounding factors. Lower limb strength has been associated with an increased risk of falls among the elderly^1^^,^ 10 ^,^ 19. Some studies have reported that fallers showed only 37% of knee extensor strength when compared to age-matched normative values^20^. In the present study, the dynamic strength of knee extensor muscles did not differ between groups (p=0.06). The lower strength observed around the knee flexor muscles in the FG is in agreement with other studies that applied dynamic maximal strength^19^ and peak isometric torque tests^10^. Reduced capacity to produce strength in the lower limbs is an important fall predictor among older adults because the capacity to recover balance depends on the magnitude and rate of joint torques that are produced. Furthermore, the moderate effect size observed for the group differences in the present study reinforces the relevance of the results.

The elderly subjects with fall history demonstrated lower minimum toe clearance during the swing phase. Reduced minimum toe clearance is an age-related gait alteration^21^ and may expose the elderly to a trip and consequently to increased risk of falling^22^. Although statistical differences were observed in the present study, the effect size magnitude was very small.

The lack of differences between groups in stride length and frequency may be explained by the low gait speed presented in this study. In fact, differences between fallers and non-fallers for stride length and frequency have been observed only at high gait velocities^23^. At high velocities, some adjustments in the gait cycle are required, resulting in reduced double support time and a greater gait instability, which may increase risk for falls^24^.

The increased time by fallers to perform the 8-foot up-and-go test may indicate dynamic balance and mobility problems. In addition, an increased time to perform the 8-foot up-and-go test was associated with greater chances of falling. Our results are in agreement with others that have demonstrated an association between poor performance in these tests and increased risk of falls^14^^,^ 25. The other functional tests did not demonstrate enough specificity to discriminate fallers and non-fallers (30-s chair-to-stand, sit-and-reach, and 6MW tests).

Although the knee extensor strength and the minimum toe clearance have been smaller in fallers than in non-fallers, these isolated parameters are not related to falls. The maintenance of balance does not depend only on the neuromuscular system, but on the integrity and interaction of several systems involved in postural control (e.g., visual and vestibular systems)^26^. Thus, the 8-foot up-and-go test requires several components such as strength to stand and sit and agility to displace and change direction and seems to reproduce better the complexity of real world conditions, where the risk of falls is greater.

A possible limitation of the present study was the classification of fallers as those who had fallen in the last 12 months, which can result in memory bias. However, the rate of fall occurrence was approximately 30% and is in agreement with the literature^1^. In addition, the loss of proprioception that occurs with aging was not assessed and may have influenced the data^27^.

## Conclusion

The present study confirmed previous findings that lower limb strength, especially knee flexor strength, is lower in the groups that experienced at least one episode of falling during the last 12 months. The lack of association between strength and falls may indicate that other aspects, such as balance and agility to recover balance and avoid a fall, may be involved.

The elderly subjects showed less minimum toe clearance, which can result in a trip and subsequent accidental fall. The data suggests that the 8-foot up-and-go test may be appropriate to identify elderly individuals with an increased risk of falling. In addition, those with a poorer performance in this test have more chances of falling. Experimental studies are required to determine other factors that could be potentially related to falls, such as strength, balance, and functionality, and that could to provide a protective effect and reduce the risk of falling.
